# Comparative performance of the InBios SCoV-2 Detect ^TM^ IgG ELISA and the in-house KWTRP ELISA in detecting SARS-CoV-2 spike IgG antibodies in Kenyan populations

**DOI:** 10.12688/wellcomeopenres.20240.2

**Published:** 2025-07-28

**Authors:** Bernadette Kutima, Eunice Wageci Kagucia, Kennedy Mwai, Makobu Kimani, Antipa Sigilai, Daisy Mugo, Henry Karanja, John N Gitonga, Angela Karani, Donald Akech, Monica Toroitich, Boniface Karia, James Tuju, Abdhalah K. Ziraba, Godfrey Bigogo, Caroline Ochieng, Clayton Onyango, Shirley Lidechi, Patrick K. Munywoki, Sophie Uyoga, Ifedayo M. O. Adetifa, Lynette I Ochola Oyier, Philip Bejon, J Anthony G Scott, Ambrose Agweyu, George M. Warimwe, James Nyagwange, Kenya SARS-CoV-2 Serology Consortium

**Affiliations:** 1 KEMRI-Wellcome Trust Research Programme, Center for Geographic Medicine Research, Coast, Kilifi, Kilifi County, P.O Box 230-80108, Kenya; 2 African Population and Health Research Center, Nairobi, Nairobi County, P.O. Box 10787, 00100, Kenya; 3 Kenya Medical Research Institute Centre for Global Health Research, Kisumu, Kisumu County, 00202, Kenya; 4 Kenya Medical Research Institute, Nairobi, Nairobi County, P.O Box 54840 00200, Kenya; 5 United States Centers for Disease Control and Prevention, Global Health Center, Division of Global Health Protection, Nairobi, 00100, Kenya; 6 Department of Infectious Diseases Epidemiology, London School of Hygiene and Tropical Medicine, London, WC1E 7HT, UK; 7 Nuffield Department of Medicine, Oxford University, Oxford, OX3 7BN, UK

**Keywords:** SARS-CoV-2, Immunoassay, IgG, Total immunoglobulin, Serology

## Abstract

**Background:**

The InBios SCoV-2 Detect™ IgG ELISA (InBios) and the in-house KWTRP ELISA (KWTRP) have both been used in the estimation of SARS-CoV-2 seroprevalence in Kenya. Whereas the latter has been validated extensively using local samples, the former has not. Such validation is important for informing the comparability of data across the sites and populations where seroprevalence has been reported.

**Methods:**

We compared the assays directly using pre-pandemic serum/plasma collected in 2018 from 454 blood donors and 173 malaria cross-sectional survey participants, designated gold standard negatives. As gold standard SARS-CoV-2 positive samples: we assayed serum/plasma from 159 SARS-CoV-2 PCR-positive patients and 166 vaccination-confirmed participants.

**Results:**

The overall agreement on correctly classified samples was >0.87 for both assays. The overall specificity was 0.89 (95% CI, 0.87–0.91) for InBios and 0.99 (95% CI, 0.97–0.99) for KWTRP among the gold standard negative samples while the overall sensitivity was 0.97 (95% CI, 0.94–0.98) and 0.93 (95% CI, 0.90– 0.95) for InBios and KWTRP ELISAs respectively, among the gold standard positive samples. In all, the positive predictive value for InBios was 0.83 (95% CI, 0.79-0.87) and 0.98 (95% CI, 0.96-0.99) for KWTRP while the negative predictive value was 0.98 (95% CI, 0.97- 0.99) and 0.97 (95% CI, 0.95-0.98) for InBios and KWTRP respectively.

**Conclusions:**

Overall, both assays showed sufficient sensitivity and specificity to estimate SARS-CoV-2 antibodies in different populations in Kenya.

## Introduction

Effective and reliable serological assays are crucial for determining population-level SARS-CoV-2 seroprevalence and may be useful in predicting protective immunity against severe disease ^1^ and informing control strategies such as primary vaccination or boosting ^2, 3^. Many SARS-CoV-2 ELISA assays have been developed and authorized for use in detecting vaccine or naturally induced antibodies. However, the assay cut-offs are usually optimized based on samples collected in high-income countries but not in Africa despite the evidence of higher cross-reactivity in the latter settings ^2^. High cross-reactivity may lead to false positives and unreliable seroprevalence estimates. In this study, we aimed to validate the InBios ELISA kit using local samples and compare it to the KEMRI-Wellcome Trust Research Programme (KWTRP) ELISA which has been optimized using both local and WHO standards and found to perform excellently ^4– 6^. KWTRP ELISA has been used extensively in Kenya to estimate seroprevalence in different populations including blood donors ^4^, healthcare workers ^7^, truckers ^8^, and residents of health and demographic surveillance systems ^9, 10^. InBios has also been used to estimate seroprevalence in both rural and urban settings in Kenya ^11– 13^. The seroprevalence estimates have been carried out by institutions under the Kenya Multi-Site Sero-surveillance (KEMIS) collaboration. A collaboration between the KEMRI-Wellcome Trust Research Programme (KWTRP), the African Population Health and Research Center (APHRC), the Kenya Medical Research Institute Centre for Global Health Research (KEMRI-CGHR), the US Centers for Disease Control and Prevention, Kenya (CDC-Kenya), the London School of Hygiene and Tropical Medicine (LSHTM) and the University of Warwick. KEMIS was formed to provide technical support for integrated demographic, clinical, and laboratory surveillance of SARS-CoV-2 to the Government of Kenya. For the comparability of data collected using both tests by KEMIS participating sites, we conducted a direct comparison of these assays. Both tests are based on the spike protein of SARS-CoV-2; the KWTRP ELISA is based on the full-length recombinant protein ^4^, while the InBios is based on spike epitopes ^14^.

**Table 1.** General characteristics of InBios and KWTRP ELISA.

Assay	InBios ^15^	KWTRP ^4^
Recombinant labelled protein	SARS-CoV-2 spike epitopes	Whole spike
Antibody detected	Immunoglobulin G	Immunoglobulin G
Methodology	Indirect ELISA	Indirect ELISA
Sample type and volume	Serum/plasma 4 µL	Serum/plasma 1µL
Turnaround time	1.5h	5.5h
Ratio calculation	Sample OD/Cut-off sample OD (>neg OD)	Sample OD/Neg OD (<0.2)
Cut-off ratio	Positive ≥1.1 Negative ≤0.9	Positive ≥2 Negative <2
Reported sensitivity	0.918 (95% CI, 0.853–0.956)	0.927 (95% CI, 0.879–0.961)
Reported specificity	0.989 (95% CI, 0.942–0.998)	0.990 (95% CI, 0.981–0.995)
Cost/sample (USD) *	17.18	13.11

*Cost based on purchase and shipment of only 25 InBios kits and no indirect costs for both assays

## Methods

InBios ELISA

The InBios assay was performed according to the manufacturer’s instructions. Briefly, test samples and three controls (positive sera, negative sera, and cut-off control sample) provided to ensure the integrity of the test and determine assay-specific threshold were diluted 1:100 by adding 4 µL to 396 µL of Sample Dilution Buffer. A total of 50 µL of the diluted controls (added in duplicate) and the test samples (added in singlets) were added onto the antigen-coated microtiter strip plates and subsequently incubated at 37°C for 1h. After washing six times with an automatic plate washer, 50 µL of Conjugate Solution was added and the plate was incubated at 37°C for 30min. The plates were washed six times and 75 µL of Liquid TMB substrate was added, developed for 20 min at room temperature and stopped with 50 µL of Stop Solution. The plates were read at 450 nm and the results were expressed as the Immunological Status Ratio (ISR) of the test sample OD to the average OD of the cut-off control. Samples were classified as positive if ISR ≥1.1 and negative if ISR ≤0.9. Test samples with an ISR <1.1 but >0.9 were retested in duplicate and the samples were considered positive with a retest ISR ≥1, Table 1.

KWTRP ELISA

This method has been described before ^4^. In brief, 2 µg/ml of whole trimeric spike protein was used to coat Nunc MaxiSorp™ flat-bottom 96-well plates (cat no 442404, Thermo Fisher Scientific) at 37 °C for 1 hour. The plates were washed 3 times using wash buffer (0.1% Tween 20 in 1X phosphate-buffered saline) followed by blocking with Blocker™ Casein (cat no 37528, Thermo Fisher Scientific) for 1 hour at room temperature. Heat-inactivated serum or plasma were diluted (1:800) in Blocker™ Casein and added to the plates, followed by a 2-hour incubation at room temperature. After washing as described above, 100 µl of horseradish peroxidase-conjugated goat antihuman IgG antibody (cat no 074–1002, KPL-SeraCare), diluted at 1:10,000 in wash buffer, was added and incubated for 1 hour at room temperature. After further washing, the plates were developed using o-phenylenediamine dihydrochloride (OPD) (cat no, P8412-100TAB, Sigma) substrate from Sigma for 10 minutes. The plates were then read at a wavelength of 492 nm. The results were expressed as the ratio of the OD value obtained from the test sample to that of the plate negative control. Samples exhibiting OD ratios ≥2 were classified as positive for SARS-CoV-2 IgG, while those with OD ratios <2 were considered negative, Table 1.

### Sample sets

**Figure 1.**
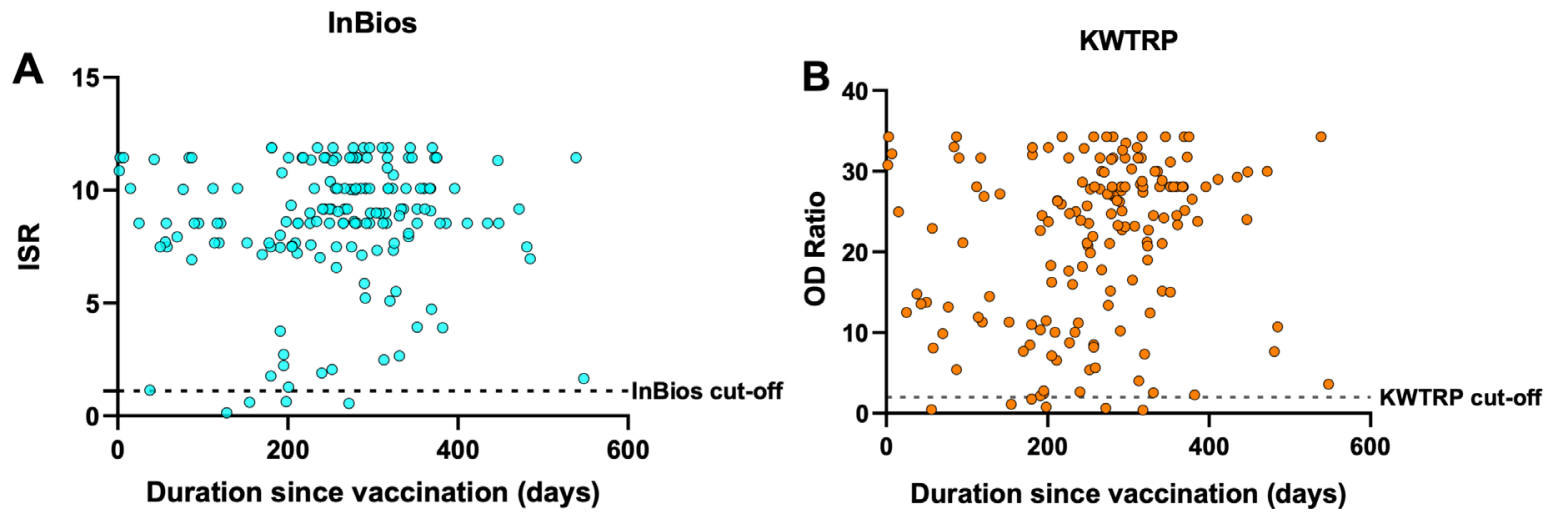
Relationship between antibody response and duration since vaccination. Each point represents one of the 166 vaccination-confirmed individuals included in the gold standard positive panel. The Immune Status Ratio (ISR) for InBios ( **A**) and Optical Density Ratio for KWTRP ( **B**) are shown for the duration between the day of vaccination and the sampling date of the serum/plasma used in the ELISAs.

The characteristics of the test populations are summarized in Table 2. The gold-standard negative samples were comprised of pre-pandemic serum/plasma that consisted of 454 adult blood donor samples collected in 2018 as part of research into the quality of transfused blood in coastal Kenya and 173 samples from annual cross-sectional surveys for malaria surveillance in coastal Kenya collected in 2018 ^4, 5^. The gold-standard positive samples were comprised of 159 COVID-19 patients sampled ≥7 days and <120 days after their PCR-positive diagnosis ^4, 5^. Additionally, we tested a pandemic panel consisting of serum/plasma from the Kilifi and Nairobi Health and Demographic Surveillance System (HDSS) sites, collected during SARS-CoV-2 serosurveys conducted in 2022 ^16^. The HDSS panel comprised 166 vaccinated participants confirmed by a vaccination certificate issued after every vaccination or a short message service (SMS) sent to their phones after every vaccination, 55 participants with a verbal report of vaccination and 573 participants unvaccinated. The median day post-vaccination was 271 (IQR = 200, 324 days) ( Figure 1).

**Table 2.** Characteristics of the test population.

Population	N	Date	Location	Participant group	Designation	Reference
Adult blood donors	454	2018	Coastal Kenya	Adults investigated for blood transfusion safety	Gold standard negatives	4
Adult cross-sectional survey	173	2018	Coastal Kenya	Adults investigated in the annual malaria cross- sectional survey	Gold standard negatives	4
SARS-CoV-2 PCR-positive cohort	159	2020	Nairobi	Adults with SARS-CoV-2 PCR positive result	Gold standard positives	4
Kilifi and Nairobi HDSS vaccination status confirmed (SMS and Certificate)	166	2022	Coastal Kenya and Nairobi	Adults and children investigated for SARS-CoV-2 seroprevalence	Gold standard positives	16
Kilifi and Nairobi HDSS vaccination status unconfirmed (Verbal report)	55	2022	Coastal Kenya and Nairobi	Adults and children investigated for SARS-CoV-2 seroprevalence	Unknowns	16
Kilifi and Nairobi HDSS unvaccinated	573	2022	Coastal Kenya and Nairobi	Adults and children investigated for SARS-CoV-2 seroprevalence	Unknowns	16

### Ethical consideration

Ethical approval for the use and collection of samples was obtained from the Kenya Medical Research Institute Scientific and Ethics Review Unit (KEMRI/SERU/CGMR- C/203/4085, 1433, 3426 and 3149) the Oxford Tropical Research Ethics Committee (44-20), and the London School of Hygiene and Tropical Medicine Research Ethics Committee (26950).

### Statistical analysis

Data analysis was conducted using GraphPad Prism v9, R v4.2.2 and Stata 15.0. Both assays were evaluated using the same sample sets to determine their specificity, sensitivity, and prevalence. To test whether there was a significant difference between InBios and KWTRP, McNemar’s test was used ^17^. We plotted ROC curves for the tests using the OD ratios of the gold standard positives and negatives to investigate whether the variations observed between the two assays were due to the assay itself or because of the selected cut-off for each assay. We assessed the assays’ reproducibility by examining the raw ODs and coefficient of variation (CV) for the negative, positive and cut-off controls for all the test runs.

## Results

InBios ELISA showed a specificity of 0.897 (95% CI, 0.871–0.919) compared to KWTRP ELISA which showed a significantly higher specificity of 0.990 (95% CI, 0.978–0.995) in the gold standard negative samples (n=627). In the gold standard positive samples, InBios and KWTRP had comparable sensitivities, 0.972 (95% CI, 0.947–0.985) and 0.935 (95% CI, 0.902–0.957) respectively. Detailed information on the sensitivity and specificity of the different sample populations is shown in Table 3. InBios reported a prevalence of 0.963 (95% CI, 0.861–0.991) and 0.898 (95% CI, 0.871–0.921), in unconfirmed (verbal report) and unvaccinated individuals, respectively while KWTRP reported a prevalence of 0.927 (95% CI, 0.817–0.973) and 0.776 (95% CI, 0.741–0.809), respectively, Table 3.

**Table 3.** InBios and KWTRP ELISA performance and prevalence of SARS-CoV-2 in the test populations.

		InBios		KWTRP	
Test Population	N	Negative	Specificity	Negative	Specificity
**Pre-pandemic samples (gold standard negatives)**	627	563	0.897	621	0.990
Adult, Coastal Kenya blood donors, 2018	454	395	0.87	449	0.989
Adult cross-sectional survey, 2018	173	168	0.971	172	0.994
	N	Positive	Sensitivity	Positive	Sensitivity
**Pandemic samples (gold standard positives)**	325	316	0.972	304	0.935
SARS-CoV-2 PCR-positive cohort	159	154	0.968	144	0.905
Kilifi and Nairobi HDSS vaccination status confirmed (SMS and Certificate)	166	162	0.975	160	0.964
	N	Positive	Prevalence	Positive	Prevalence
**Test samples (unknowns)**	628	568	0.904	496	0.790
Kilifi and Nairobi HDSS vaccination status unconfirmed (Verbal report)	55	53	0.963	51	0.927
Kilifi and Nairobi HDSS unvaccinated	573	515	0.898	445	0.776

McNemar's test showed high overall agreement between InBios and KWTRP ( Table 4). The positive predictive value (PPV) for InBios was 0.83 (95% CI, 0.79-0.87) which was relatively low compared to 0.98 (95% CI, 0.96-0.99) for KWTRP while the negative predictive values (NPV) were comparable with 0.98 (95% CI, 0.97- 0.99) and 0.97 (95% CI, 0.95-0.98) for InBios and KWTRP respectively. Because of its lower specificity, InBios resulted in more false positives – 55/454 (12.99%) and 4/173 (2%) – than KWTRP – 1/454 (0.2%) and 0/173 – among the blood donor and cross-sectional survey samples, respectively. Whereas both assays classified ≤2.5% as false negatives in the positive gold standards, KWTRP resulted in more false negatives, 11/159 (6.9%) among the PCR-positive cohort and 3/166 (1%) among the confirmed vaccinated individuals ( Table 4). The false negatives among the vaccination-confirmed cohort were not dependent on the time elapsed since vaccination for both assays ( Figure 1). Overall, InBios yielded more positive results than KWTRP in the test samples, reflecting its slightly higher sensitivity and lower specificity when compared to KWTRP.

**Table 4.** Pairwise comparison of InBios and KWTRP ELISA on the different sample sets.

			KWTRP		
	N	InBios	Positive	Negative	P-value *
**Gold standard negatives**					
Adult, Coastal Kenya blood donors, 2018	454	**Positive**	4 (0.008)	55 (0.121)	0.0000
		**Negative**	1 (0.002)	394 (0.877)	
Adult Malaria Cross-sectional Survey, 2018	173	**Positive**	1 (0.005)	4 (0.023)	0.0455
		**Negative**	0 (0)	168 (0.977)	
**Gold standard positives**					
SARS-CoV-2 PCR-positive cohort, 2020	159	**Positive**	143 (0.899)	11 (0.069)	0.0063
		**Negative**	1 (0.006)	4 (0.025)	
Kilifi and Nairobi HDSS vaccination status confirmed (SMS and Certificate), 2022	166	**Positive**	159 (0.958)	3 (0.01)	0.6250
		**Negative**	1 (0.006)	3 (0.01)	
**Test samples (unknowns)**					
Kilifi and Nairobi HDSS vaccination status unconfirmed (Verbal report), 2022	55	**Positive**	51 (0.927)	2 (0.036)	0.5000
		**Negative**	0 (0)	2 (0.036)	
Kilifi and Nairobi HDSS unvaccinated, 2022	573	**Positive**	437 (0.763)	78 (0.136)	0.0000
		**Negative**	8 (0.014)	50 (0.087)	

*McNemar's Chi-square test P-value.

**Figure 2.**
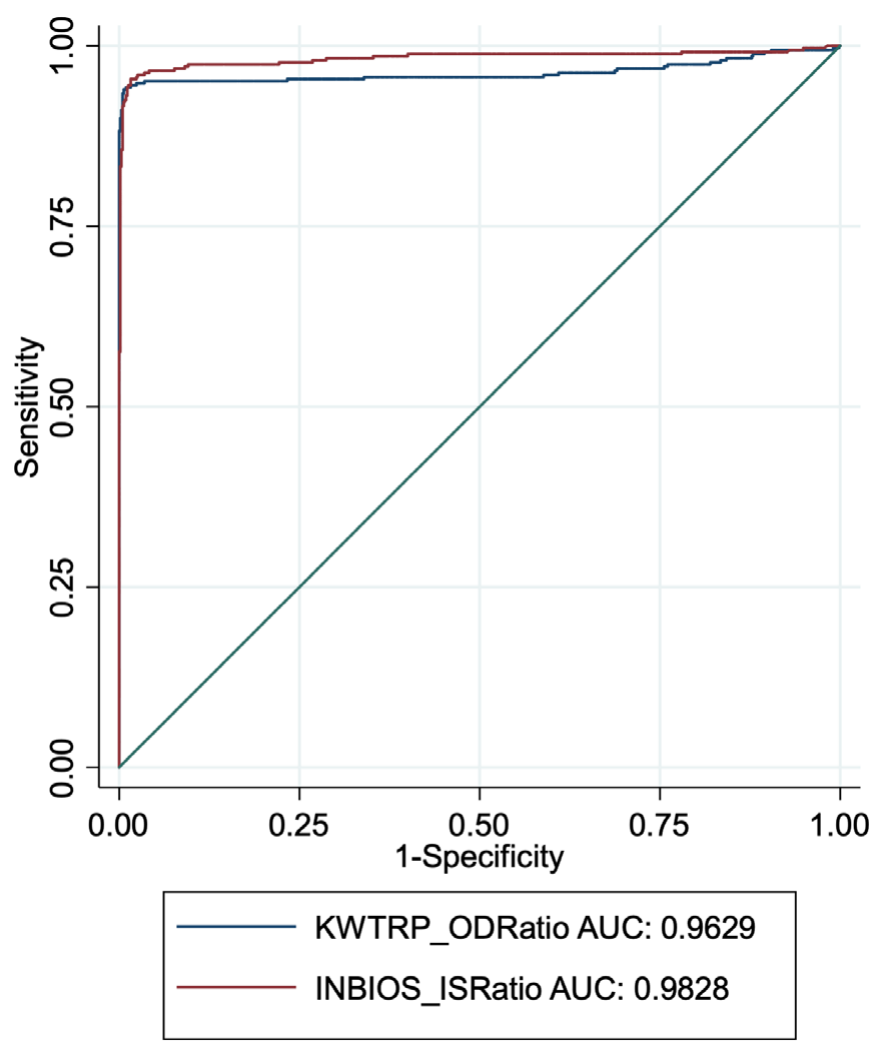
ROC curves for InBios and KWTRP ELISAs using the OD ratios of all the gold standard positives and negatives as assay population.

We plotted ROC curves for InBios and KWTRP using all the gold standard positives and negatives. The InBios curve was marginally closer to the optimal point with an area under the curve (AUC) of 0.983, slightly better than an AUC of 0.963 for KWTRP, Figure 2.

**Figure 3.**
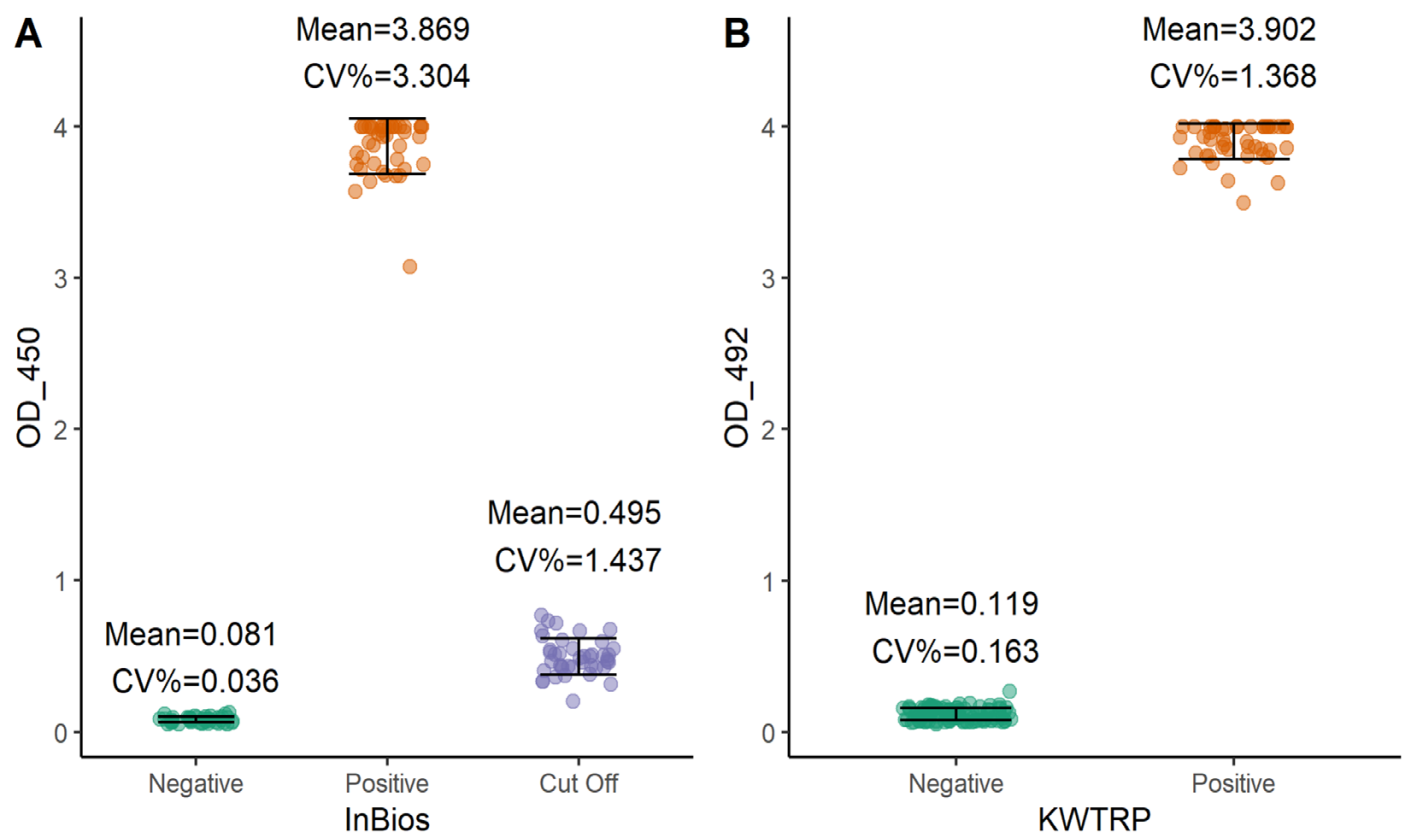
Inter-assay variation of InBios ( **A**) and KWTRP ( **B**) ELISAs by assessing the raw ODs of the negative, positive, and cut-off controls for all the runs done during the comparison.

We finally examined the performance of both assays by determining the coefficient of variation (CV) of the negative and positive controls and cut-off control in the case of InBios ( Figure 3). All the CVs were within acceptable ranges ^18^. InBios negative, positive, and cut-off controls showed CVs of 0.036%, 3.304% and 1.437%, respectively, while KWTRP negative and positive controls had CVs of 0.163% and 1.368% respectively.

## Discussion

Having standardized tests for seroprevalence studies is essential in determining pathogen exposure levels in the population ^19, 20^. Consistency in data interpretation to reduce variability enables reliable comparison, identifies risk populations to enable better targeted public health interventions and enables better tracking of changes in immunity over time within regions ^21, 22^. With this in mind we have compared two assays used extensively in Kenya to estimate SARS-CoV-2 seroprevalence with the same set of samples to enable comparability of the results within the country. Overall, InBios showed higher sensitivity than KWTRP which in turn showed higher specificity ( Table 3). This resulted in a higher number of positive samples across all the panels in the table tested by InBios than KWTRP, including false positives in the pre-pandemic sample set. The false positives imply that InBios, with a specificity of 89.2%, would overestimate seroprevalence when true positives are in the test population and possibly miss the waning of antibodies when the seroprevalence is low. The seroprevalence observed was a result of the COVID-19 vaccination rollout that began in March 2021, as well as by natural infection through the four COVID-19 waves occurring since May 2021, that resulted from the Delta variant, Omicron BA.123, Omicron BA.4/5, and Omicron BQ.1 variant is expected to remain high following (re)infection and vaccination which cannot be distinguished by the tests. Therefore, under these circumstances where seroprevalence is high, InBios would better estimate true seroprevalence than KWTRP. However, both assays would still underestimate the true seroprevalence in the context of high SARS-CoV-2 seroprevalence. This underscores the importance of statistical adjustment for test performance – as has been conducted for previous serosurveys to yield more accurate estimates of true seroprevalence ^4, 5, 7, 24, 25^. 

Compared to its reported sensitivity of 91.8% during development, InBios reported a lower sensitivity of 83.2% from a sampled population in India ^14^. In contrast, we observed a higher sensitivity of ≥ 96.8% using our gold standard positive sample sets. In our gold standard negatives panel, InBios’s specificity was 89.2%% compared to its reported specificity of 98.9% during development and ≥96.7% in a sampled population in India ^14^. These differences illustrate the importance of assay validation and cut-off determination using samples from diverse geographical regions. The lower InBios specificity observed in our setting may partly be due to threshold selection along ROC curves as many false positives were closer to the cut-off ( Figure 2). Higher cross-reactivity may have also played a role in the high numbers of false positives, and there are several reports of higher SARS-CoV-2 cross-reactivity in African samples ^2, 26– 30^. The lower sensitivity of KWTRP in the PCR-positive cohort has been reported previously and was driven by several samples collected <28 days after testing positive by PCR ^4^.

A limitation of this study is the use of convenient cross-sectional samples from vaccinated people as gold standard positives since they limit the interpretation of the antibody responses observed in Figure 1. This is because it’s impossible to account for boosting due to natural infection and may explain why there was no decline in response several months post-vaccination in both assays ( Figure 1). Overall, both assays showed low CV% implying very high reproducibility. With adjustment for test performance, both assays provide sufficient tools in estimating the seroprevalence of vaccine and naturally induced SARS-CoV-2 IgG antibodies within a sub-Saharan African setting and including populations sampled in the context of recently circulating variants.

## Disclaimer

The findings and conclusions in this study are those of the authors and do not necessarily represent the official position of the U.S. Centers for Disease Control and Prevention.

## Data Availability

Harvard Dataverse: Underlying data for ‘Comparative performance of the InBios SCoV-2 DetectTM IgG ELISA and the in-house KWTRP ELISA in detecting SARS-CoV-2 spike IgG antibodies in Kenyan populations’, https://doi.org/10.7910/DVN/FOYVMT ^23^. Harvard Dataverse: Extended data for ‘Comparative performance of the InBios SCoV-2 DetectTM IgG ELISA and the in-house KWTRP ELISA in detecting SARS-CoV-2 spike IgG antibodies in Kenyan populations.’ https://doi.org/10.7910/DVN/FOYVMT ^23^. This project contains the following extended data: BKutima_DATASET_Codebook_31072023.pdf BKutima_DATASET_Readme_31072023.txt datasets.zip Inbios_KWTRP_230523.do InBios_KWTRP_coefficient of variation plots.R InBios_KWTRP_vaccinated_ELISA_21062023.pzfx KWTRP_Inbios ROC.do Data are available under the terms of the Creative Commons Attribution 4.0 International license (CC-BY 4.0)
