# Supplementary material for: Comparative performance of the InBios SCoV-2 Detect TM IgG ELISA and the in-house KWTRP ELISA in detecting SARS-CoV-2 spike IgG antibodies in Kenyan populations
Source: Wellcome Open Res. 2025 Jul 28;9:349. Originally published 2024 Jun 28. [Version 2] doi: 10.12688/wellcomeopenres.20240.2 (PMC12368485; doi:10.12688/wellcomeopenres.20240.2)
Supplement: Peer review history

# Reviewer report: Reviewer response for version 2

**DOI:** 10.21956/wellcomeopenres.27114.r127789

**Published:** 2025-08-20

**Authors:** Samira Bührer-Sékula

**Affiliations:** 1 Universidade Federal de Goiás-Merck, Goiânia, GO, Brazil

I have carefully reviewed the revised version of the manuscript “Comparative performance of the InBios SCoV-2 Detect™️ IgG ELISA and the in-house KWTRP ELISA in detecting SARS-CoV-2 spike IgG antibodies in Kenyan populations.”

 I acknowledge that the authors have adequately addressed the main concerns raised in my previous review, particularly the inclusion of PPV and NPV, the provision of catalogue numbers for reagents, and the expanded discussion on assay performance, emerging variants, and regional harmonization. While not all of my initial suggestions were incorporated, the revisions made have significantly improved the clarity and scientific rigor of the manuscript.

 Therefore, I am satisfied with the current version and would like to update my recommendation to Approved.

Is the work clearly and accurately presented and does it cite the current literature?

Yes

If applicable, is the statistical analysis and its interpretation appropriate?

Yes

Are all the source data underlying the results available to ensure full reproducibility?

Yes

Is the study design appropriate and is the work technically sound?

Yes

Are the conclusions drawn adequately supported by the results?

Partly

Are sufficient details of methods and analysis provided to allow replication by others?

Yes

Reviewer Expertise:

NA

I confirm that I have read this submission and believe that I have an appropriate level of expertise to confirm that it is of an acceptable scientific standard.

# Reviewer report: Reviewer response for version 2

**DOI:** 10.21956/wellcomeopenres.27114.r127788

**Published:** 2025-07-29

**Authors:** Abhinay Gontu

**Affiliations:** 1 The Pennsylvania State University, University Park, Pennsylvania, USA

Thanks you for the update on the manuscript. The authors have addressed some of my comments like the inclusion of predictive values and some parts of discussion. The paper could have been improved by not over stating some points like the use of the term "gold-standards" throughout the paper and "excellently" might turn to mislead some readers. Over all, it could have been improved better.

Is the work clearly and accurately presented and does it cite the current literature?

Partly

If applicable, is the statistical analysis and its interpretation appropriate?

Partly

Are all the source data underlying the results available to ensure full reproducibility?

Yes

Is the study design appropriate and is the work technically sound?

Partly

Are the conclusions drawn adequately supported by the results?

Partly

Are sufficient details of methods and analysis provided to allow replication by others?

Yes

Reviewer Expertise:

Immunoassays, vaccines & therapeutics

I confirm that I have read this submission and believe that I have an appropriate level of expertise to confirm that it is of an acceptable scientific standard.

# Reviewer report: Reviewer response for version 1

**DOI:** 10.21956/wellcomeopenres.22415.r92582

**Published:** 2024-08-27

**Authors:** Abhinay Gontu

**Affiliations:** 1 The Pennsylvania State University, University Park, Pennsylvania, USA

The article compares two indirect format ELISAs, the InBios SCoV-2 DetectTM IgG ELISA (InBios) and the in-house KWTRP ELISA (KWTRP), aiming to assess the comparability of data for SARS-CoV-2 seroprevalence in the Kenyan population. The authors report an overall agreement of >0.87 between the assays and primarily rely on sensitivity and specificity to determine the suitability of these assays. They conclude that both assays could be used for seroprevalence testing in Kenya.

 While the article is well-designed, with figures illustrating parts of the results, I have several suggestions that could enhance the study's impact for a broader audience:

**Major Points:**

1. The terms “gold positive” and “gold negative” should be revised throughout the paper. The samples used for validation or comparison were simply earlier PCR-confirmed samples (for the presence of SARS-CoV-2 antibodies) or pre-pandemic samples.
2. How were the confirmed positives selected? Were they derived from a cohort that was PCR-positive or vaccinated? Was any additional confirmatory test, such as virus neutralization, performed to verify the presence of antibodies? If not, this should be highlighted as a significant limitation of the study.
3. In studies comparing assays, including a 2x2 contingency table helps readers understand the comparison clearly. I recommend adding two 2x2 tables: one for previously confirmed samples and another for the test set used in this study.
4. While sensitivity and specificity are provided, in a population setting, predictive values (positive and negative) offer a more comprehensive understanding of the assays' performance. A relevant example can be found in this study: https://journals.plos.org/plosone/article?id=10.1371%2Fjournal.pone.0246346
5. The ELISA antigens are mentioned as the spike protein/spike epitopes. Could you specify which variant's spike protein was used? The assays' performance could vary depending on the presence of different circulating SARS-CoV-2 variants and the corresponding antibodies. Are multiple vaccines being used? If so, which ones? It might be useful to stratify the population based on timelines or vaccine types.
6. Figure 1 mentions the vaccinated population, yet no trend over time is observed. Could this data be stratified by days post-vaccination to better understand where the ELISAs were effective and where they were not? The persistence of high antibody levels at 600 days is surprising. Could this indicate reinfection? Such points could be explored further in the discussion.
7. Does the time elapsed after PCR confirmation affect the performance of antibody assays? This could be represented in a figure for clarity.
8. The ISR values of InBios and the OD ratios of KWTRP vary significantly. In InBios, there’s a large cluster of samples above 5, while KWTRP shows a random spread. What is the correlation between these two outputs?

**Minor Points:**

1. There is currently no defined antibody correlate of protection for SARS-CoV-2, yet the authors suggest that seroprevalence can predict protective immunity.
2. More references are needed to support the evidence for high cross-reactivity. Why is this occurring? Are there specific test conditions or the presence of endemic coronaviruses influencing this?
3. The KWTRP ELISA was tested using WHO standards. Are these standards available for validation by others? What are their characteristics? The term "performed excellently" is vague; can this performance be quantified?
4. Why weren’t the cut-offs for both assays based on screening pre-pandemic samples, especially for InBios? Given the high number of false positives, would the authors recommend a different cut-off?
5. Catalog numbers are mentioned inconsistently—please ensure uniformity in reporting.
6. Were the ELISAs validated for inter-assay and intra-assay variance?
7. The authors discuss prevalence rates, reinfection, and ELISA sensitivity. It would be beneficial to discuss these predictive values in the context of disease prevalence.
8. Has InBios been used in other regions beyond the one mentioned in India? If yes, what were the results?

Is the work clearly and accurately presented and does it cite the current literature?

Partly

If applicable, is the statistical analysis and its interpretation appropriate?

Partly

Are all the source data underlying the results available to ensure full reproducibility?

Yes

Is the study design appropriate and is the work technically sound?

Partly

Are the conclusions drawn adequately supported by the results?

Partly

Are sufficient details of methods and analysis provided to allow replication by others?

Yes

Reviewer Expertise:

Immunoassays, vaccines & therapeutics

I confirm that I have read this submission and believe that I have an appropriate level of expertise to confirm that it is of an acceptable scientific standard, however I have significant reservations, as outlined above.

# Reviewer report: Reviewer response for version 1

**DOI:** 10.21956/wellcomeopenres.22415.r91219

**Published:** 2024-08-08

**Authors:** Samira Bührer-Sékula, Matheus Bernardes Torres

**Affiliations:** 1 Universidade Federal de Goiás-Merck, Goiânia, GO, Brazil; 2 Instituto de Patologia Tropical e Saúde Pública, Universidade Federal de Goias, Goiânia, State of Goiás, Brazil

The article titled "Comparative performance of the InBios SCoV-2 DetectTM IgG ELISA and the in-house KWTRP ELISA in detecting SARS-CoV-2 spike IgG antibodies in Kenyan populations" presents a direct comparison of two ELISA assays to estimate SARS-CoV-2 seroprevalence in Kenya. The study evaluates the sensitivity, specificity, and overall performance of the InBios and KWTRP assays using local serum/plasma samples collected pre- and post-pandemic. The results indicate that while both assays are comparable and suitable for estimating seroprevalence, InBios exhibits slightly higher sensitivity, whereas KWTRP shows higher specificity.

 Overall, the study presents valuable insights into the performance of two ELISA assays for detecting SARS-CoV-2 antibodies in Kenyan populations. While the manuscript is well-written and the study design is robust, addressing minor issues will enhance the clarity and strengths of the research. The authors are commended for their comparative analysis but should improve the discussion section, which will contribute to the field of SARS-CoV-2 serology.

 Points to be considered:

1. The last phrase of the introduction should be in the methods section.
2. Figure 1 shows the characteristics of both tests and should be cited in the methods section.
3. Use consistent order when citing the assays "InBios first and KWTRP second for example", please revise the order in the manuscripts, figures and tables.
4. Suggestion: change “positive gold standard samples” and “negative gold standard samples” to "confirmed positive samples" and "negative control samples".
5. If available, please provide details on the specific epitope regions (amino acid positions) utilized in the InBios assay as it helps to precisely identify which parts of the protein are involved.
6. Which COVID-19 vaccines were administered in Kenya? Do you have data on which vaccines the study participants received? Do the vaccines used in Kenya contain both the S and N proteins, or only one of these proteins? Could an additional N protein ELISA differentiate between the serological responses of vaccinated and unvaccinated individuals to SARS-CoV-2? Please, complement the discussion section.
7. What is the accuracy of the tests? Please include this information in the results section. Current data suggest that the InBios test may have lower accuracy in detecting anti-SARS-CoV-2 antibodies. It is important to note that studies with larger sample panels have demonstrated that some COVID-19 patients do not seroconvert, not producing antibodies against the pathogen ( for example, see Fogaça MBT *et al (*2024 ^1^ ) **; Harritshøj LH *et al(*2021 ^2^ ); Haselmann V et al (2020 ^3^ ) ).
8. The manuscript asserts that "InBios would better estimate true seroprevalence than KWTRP in high seroprevalence settings." Could you elucidate why InBios would be preferable, given that it yields significantly more false-positive results compared to KWTRP (Table 4), including among individuals with other endemic infectious diseases such as malaria?
9. InBios ELISA, despite not using the whole trimeric protein, presents higher false positive rates compared to KWTRP ELISA. Could you suggest reasons for this unexpected result? Are there specific antigenic regions, cross-reactivity issues, or assay cut-off thresholds that might explain these higher false positive rates?
10. In the discussion section, it would be interesting to include an analysis of the turnaround time and cost per sample (USD) for both tests. How did you determine the cost per sample? Did you calculate only the reagent costs, or did you include the entire process, including labor and time costs?
11. Discuss the importance of having similar tests for better seroprevalence comparison studies in the same continent/ epidemiological area.
12. Discuss the high seroprevalence of the unknown samples. Was it expected? Comment prevalence of COVID during collection of samples in the study area.

Is the work clearly and accurately presented and does it cite the current literature?

Yes

If applicable, is the statistical analysis and its interpretation appropriate?

Yes

Are all the source data underlying the results available to ensure full reproducibility?

Yes

Is the study design appropriate and is the work technically sound?

Yes

Are the conclusions drawn adequately supported by the results?

Partly

Are sufficient details of methods and analysis provided to allow replication by others?

Yes

Reviewer Expertise:

Development of diagnostic tests based on ELISA and lateral flow immunoassay.

We confirm that we have read this submission and believe that we have an appropriate level of expertise to confirm that it is of an acceptable scientific standard, however we have significant reservations, as outlined above.

## References

[1] : Production of antigens expressed in Nicotiana benthamiana plant and Escherichia coli for the SARS-CoV-2 IgG antibody detection by ELISA. *J Virol Methods* .2024;329: 10.1016/j.jviromet.2024.114969 114969 10.1016/j.jviromet.2024.114969 38834144

[2] : Comparison of 16 Serological SARS-CoV-2 Immunoassays in 16 Clinical Laboratories. *J Clin Microbiol* .2021;59(5) : 10.1128/JCM.02596-20 10.1128/JCM.02596-20 33574119 PMC8091860

[3] : Comparison of test performance of commercial anti-SARS-CoV-2 immunoassays in serum and plasma samples. *Clin Chim Acta* .2020;510: 10.1016/j.cca.2020.07.007 73-78 10.1016/j.cca.2020.07.007 32652161 PMC7343640
